# Tracing the Trans-Pacific Evolutionary History of a Domesticated Seaweed (*Gracilaria chilensis*) with Archaeological and Genetic Data

**DOI:** 10.1371/journal.pone.0114039

**Published:** 2014-12-11

**Authors:** Marie-Laure Guillemin, Myriam Valero, Sylvain Faugeron, Wendy Nelson, Christophe Destombe

**Affiliations:** 1 Instituto de Ciencias Ambientales y Evolutivas, Facultad de Ciencias, Universidad Austral de Chile, Casilla 567, Valdivia, Chile; 2 CNRS, Sorbonne Universités, UPMC University Paris VI, UMI 3614, Evolutionary Biology and Ecology of Algae, Station Biologique de Roscoff, CS 90074, Place G. Tessier, 296888 Roscoff, France; 3 Centro de Conservación Marina and CeBiB, Facultad de Ciencias Biológicas, Pontificia Universidad Católica de Chile, Santiago, Chile; 4 National Institute of Water & Atmospheric Research, School of Biological Sciences, University of Auckland, Private Bag 14901, Wellington 6241, New Zealand; College of Charleston, United States of America

## Abstract

The history of a domesticated marine macroalga is studied using archaeological, phylogeographic and population genetic tools. Phylogeographic and population genetic analyses demonstrated that the cultivated red alga *Gracilaria chilensis* colonised the Chilean coast from New Zealand. Combining archaeological observations with phylogeographic data provided evidence that exchanges between New Zealand and Chile have occurred at least before the Holocene, likely at the end of the Last Glacial Maximum (LGM) and we suggest that migration probably occurred via rafting. Furthermore, the remarkably low microsatellite diversity found in the Chilean populations compared to those in New Zealand is consistent with a recent genetic bottleneck as a result of over-exploitation of natural populations and/or the process of domestication. Therefore, the aquaculture of this seaweed, based essentially on clonal propagation, is occurring from genetically depressed populations and may be driving the species to an extinction vortex in Chile.

## Introduction

Humans have always exploited species of plants and animals. Although archaeological evidence has shown the natural environment was altered by hunter-gatherers, the major impact of human activities on biodiversity is considered to have begun when humans started the domestication process, over 13,000 years ago, at the beginning of the Holocene period [1]. In turn, human cultivation practices during the Neolithic period considerably modified human evolution in such a way that domestication of plants can be considered as the end result of a long co-evolutionary continuum [2]. The process of domestication has shaped terrestrial biodiversity, reducing gene diversity and modifying life history traits of a variety of land plants [3], [4], [5] and animal species [6]. It is only recently that archaeological studies, in conjunction with ecological approaches, have highlighted the interactions of humans with marine biodiversity [7]. The most ancient evidence of coastal foraging was uncovered in South African Middle Stone Age archaeological sites (≈160,000 years) [8]. Several authors have suggested that marine biodiversity probably played a central role for subsistence in harsh environmental conditions during periods of glaciation because of the buffering effect of the sea against climatic variations on land [9]. In particular, seaweed beds and their associated biodiversity were considered to be major determinants of the human colonisation route from northern Asia to America during the Last Glacial Maximum (LGM), as proposed by the Kelp Highway Hypothesis [10]. These authors suggested that kelp forests, by providing shelter and food, allowed the establishment of human populations along the Pacific American coast before their migration inland and the subsequent development of agriculture. The uncovering of remnants of eleven species of algae, including partially burned or squashed fragments on stone tools in the Monte Verde site in South Chile attests that human exploitation and consumption of seaweeds began at least 14,600 years ago [11]. In addition, these authors proposed that inland Paleo-amerindian populations might have used some of these coastal species as goods for trading. However, the effects of human pressure on marine biodiversity were not the main focus of these previous studies, and it is only recently that the huge impact of early human settlements on intertidal and nearshore habitats has been examined [7].

Domestication of marine species appears very recent compared to terrestrial examples. The earliest tentative record was dated around 600y BP but the vast majority of marine species were domesticated in recent decades [12]. Thus, human effects on marine biodiversity via domestication are mostly linked to the industrial revolution. Taken together with massive overexploitation of natural stocks, these effects are contributing to the sixth mass extinction phenomena [13]. The consequences of domestication have the potential to severely decrease species genetic diversity when combined with particular cultivation practices, such as clonal propagation [14], [15], [16]. Overall, human impacts on marine biodiversity are a combination of processes (fishing/harvesting, management, cultivation, and domestication), and the relative influence of these processes has been evolving over the past millennia.

Here we consider the case of the red alga *Gracilaria chilensis* Bird, McLachlan et Oliveira and analyse the historical interaction between humans and seaweeds. Archaeological evidence suggests that this species was used for food and/or medicine by the first inhabitants of Monte Verde in Southern Chile, about 14,600 years before present [11], and that primary, marine-adapted economies developed along the South Pacific coast during the early and middle Holocene period [17]. From the 1970s, natural beds of *G. chilensis* were extensively harvested in Chile for agar production, until they collapsed in the late 1980s because of overexploitation [18]. This crisis coincides with a rather limited genetic diversity [14]. Today, *G. chilensis* is by far the most important seaweed crop cultivated in Chile [18], and also one of the few seaweeds for which evidence of domestication has been reported [14]. Populations of this species are found in estuaries where individuals are free-floating and reproduce asexually, and also on rocky shores where the species reproduces sexually with an alternation of haploid and diploid individuals attached to the substratum [14]. Both ancestral, extensive culture practices and intensive aquafarming are based on vegetative propagation, using thallus cuttings and re-planting onto muddy soft sediments in bays and estuaries [19]. In small traditional farms, algal fragments set adrift during manual harvesting were shown to contribute to re-seeding these beds [20]. While *G. chilensis* displays a disjoint, trans-oceanic distribution range [21], and there have been attempts to explore the commercial use of *Gracilaria* in Australia and New Zealand [22], [23], no significant harvesting or farming has been reported in this southwest Pacific region. This situation offers the opportunity to investigate potential changes in genetic diversity associated with different levels of historical and contemporary human activities.

In this study we retrace the evolutionary history of *G. chilensis* on both sides of the Pacific Ocean to better understand the state of the species genetic resources in Chile, a region where the species has faced long-term extensive exploitation and recent domestication. We first investigate the history of the disjoint distribution through a phylogeographic approach. In particular, we test the hypothesis of a range extension from New Zealand through trans-Pacific dispersal. We also investigate potential human impacts on genetic diversity using population genetic analyses in an attempt to resolve the recent history of this domesticated species.

## Materials and Methods

### Sampling

Nineteen populations were sampled in New Zealand and in Chile covering the disjoint range distribution of the species (Table S1): 187 tetrasporophytes (diploid individuals) were collected from eight natural populations from New Zealand (including the Chatham Islands) and 410 diploid individuals from 11 populations collected along the Chilean coast were gathered from a published data set [14]. No specific permissions were required for sampling in the 11 locations were *Gracilaria chilensis* was extracted in Chile. For the eight populations from New Zealand, collections were made under a Special Permit to NIWA from the New Zealand Ministry of Primary Industries. GPS coordinates of all locations are available in the Table S1. Field studies did not involve sampling of endangered or protected species. Details for the location and sample size of the study populations are given in Table S1. The ploidy level of the individuals was determined by observations of the reproductive organs under a binocular microscope. Voucher herbarium specimens from New Zealand are deposited in WELT [24].

### Genotyping and Sequencing

DNA extraction followed [25] and PCR amplification of the five microsatellite loci and allele size scoring were performed according to [26]. Products were run on 6.5% polyacrylamide denaturing gels in a LI-COR DNA sequencer model 4200TM (LI-COR, Lincoln, NE, USA). A total of 93 individuals from New Zealand and 108 individuals from Chile were sequenced for the Internal Transcribed Spacer 2 (ITS2, operon of nuclear ribosomal genes). Eleven individuals (three from Chile and six from New Zealand) were sequenced for the ribulose 1,5-bisphosphate carboxylase/oxygenase gene (*rbc*L, plastid) and compared with two sequences of *G. chilensis* from Chile already deposited in GenBank: AY049396 from Coquimbo [27] and DQ095784 from Lachangua [28]. The ITS2 PCR reactions and sequencing followed the protocol described in [25]. The *rbc*L amplifications were carried out following the procedures described by [14]. Sequences of 481 bp of ITS2 (GENBANK N° HQ998639 to HQ998839) and of 641 bp of *rbc*L (GENBANK N° HQ998840 to HQ998848) were obtained with an ABI 3100 Sequencer (Applied Biosystem, Foster City, CA). Sequences were edited using CHROMAS [29] and multiple sequence alignments were constructed with BIOEDIT [30].

### Data Assessment

To minimise the rate of genotyping errors, a second and third round of PCR and electrophoresis were performed for individuals with dubious multilocus genotypes (i.e. with missing data or displaying rare alleles). Because of the very high amplification failure rate (almost 0.3) of the locus Grc-AC/CT23 [26] in the three populations from the western part of New Zealand (i.e. NZ-PGB, NZ-MOU and NZ-WIN), probably linked to very high proportion of null alleles, this locus was not included in this study.

### Data Analysis

For nuclear microsatellites loci, expected heterozygosity (He), observed heterozygocity (Ho), allele number and the number of private alleles were calculated in each population using FSTAT v 2.9.3 [31]. *F*_IS_ was calculated over all loci according to [32] using FSTAT v 2.9.3 [31]. Deviation from Hardy–Weinberg equilibrium and linkage disequilibrium were tested in each population and over all samples by using 5000 permutations and Bonferroni correction with FSTAT v 2.9.3 [31]. For ITS2 sequences, the number of ribotypes (nR) were computed using ARLEQUIN v 3.11 [33]. Allelic richness (Ae) and ribotypic richness (Re) were estimated using the rarefaction procedure of Petit et al. [34] implemented in CONTRIB, which takes into account differences in sample size. These estimates were calculated in each population with a rarefaction size of 20 genes for microsatellites (corresponding to the number of allele in the smallest diploid population, NZ-PGB, N = 2×10) and 5 ribotypes for the ITS2 (smallest population, NZ-CAH, N = 5). We calculated allele accumulation over sampling size for both Chile and New Zealand (including Chatham Island) by jackknife resampling (100 times), using the package PopGenKit v1.0 [35] implemented in R v3.0.3 (http://cran.r-project.org/). We applied sample size intervals of one unit, varying from one to a maximum of 410 individuals for Chile and a maximum of 187 individuals for New Zealand.

A median-joining network of ITS2 ribotypes was constructed for both the New Zealand and Chilean data sets using NETWORK v 4.5 [36].

To compare population structure in New Zealand and Chilean populations using microsatellites, we employed two different methods. First we used a Bayesian, model-based genetic admixture analysis implemented in STRUCTURE [37]. Individuals were combined into one dataset for analysis, without any a priori population assignments and admixture was allowed. Each number of assumed populations (K, set sequentially from 1 to 11) was run six times, using the admixture model, independent allele frequencies, λ = 1, with a burn-in of 50,000 iterations and a run-length of 50,000 iterations. The “true” number of K was inferred both from the posterior probability of the data Ln(*p*). We ran STRUCTURE 50 times at K = 5 according to the highest Ln(*p*) in the previous set of runs, and combined the result of all these runs with the programme CLUMMP [38] and visualised this combined data with DISTRUCT [39]. Second, we used the programme POPULATION GRAPH that uses a graph theory approach, to determine the topological relationship among populations that may currently be exchanging genes [40]. This method is free of a priori assumptions about geographic arrangements of sampled populations and works by simultaneously determining the high-dimensional covariance relationships among all populations using genetic marker data. The programme, then, determines the minimum set of edges (connections) that sufficiently explain the among-population covariance structure of all of the populations. The network of population connections can then be analysed by various *post hoc* analyses. POPULATION GRAPH was implemented on the web (http://dyerlab.bio.vcu.edu/wiki/index.php/) using the population genetic dataset. A test for distinct clustering of New Zealand and Chilean populations was conducted *a posteriori* using the methods outlined by Dyer and Nason [40].

To detect bottlenecks, we used the graphical method developed by Luikart et al [41] by grouping alleles of the five microsatellite loci into 10 allele’s frequency classes. Luikart et al. [41] argued that due to the probabilistic reduction of rare alleles, bottlenecks might cause a characteristic mode-shift distortion in the typical L-shape distribution of allele frequencies, i.e. low number of low-frequency alleles. Moreover, the magnitude and timing of past changes in population size was inferred from the analysis of ITS2 mismatch distributions with ARLEQUIN v 3.11 [33]. Mismatch distribution were calculated for the whole ITS2 data set and then for several partitioning of the ITS2 data set: (Chile, New Zealand, Western New Zealand, Eastern New Zealand (excluding Chatham Island) and Chatham Island alone). First, Fu’s Fs [42] and Ramos-Onsins and Rozas’s [43] R_2_ statistic were estimated and significant departure from mutation-drift equilibrium was tested by 10,000 bootstrap replicates using DnaSP version 5.10.01 [44]. Second, the mismatch distribution of each partition was compared to a distribution expected under a model of spatial population expansion by calculating the sum of squared differences between observed and expected distributions and the Harpending’s raggedness index [45]. Significance was assessed by 10,000 bootstraps. We calculated the intra-population coalescence time (i.e. time since the start of a population expansion) from the statistic τ = 2ut, where t is the number of years since a population expansion and u is the per-sequence-per-year mutation rate. We used ITS2 mutation rates proposed by Koch et al. [46] in plants: an average of 1.4%, varying from 0.5 to 2.5%.

## Results

### Genetic diversity

**Figure 1:**
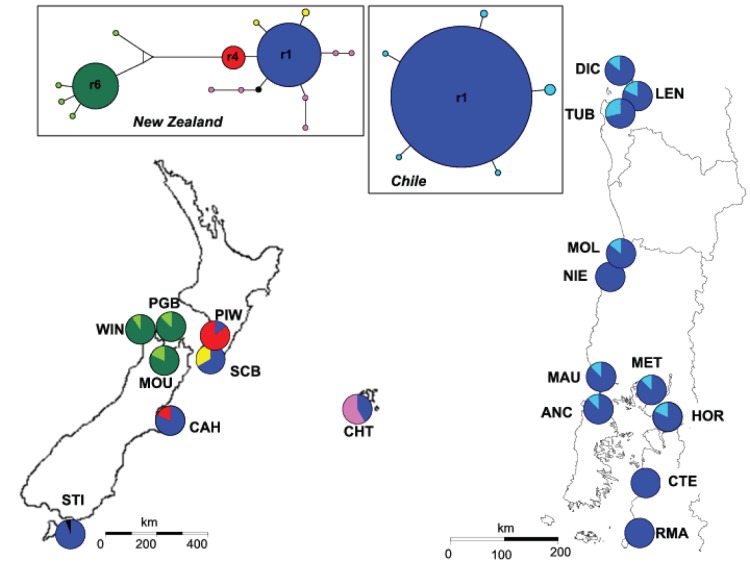
Location of the sampling sites. ITS2 ribotype distribution and ITS2 network including 201 sequences of 481 nucleotides. Pie charts represent ribotype frequency in each population. Ribotype network was constructed using a median-joining algorithm with programme NETWORK [36]. Colours of the network correspond to those mapped with rare ribotypes in each “region” pooled under the same colour for clarity. Circle sizes are proportional to total ribotype frequency and connection lengths correspond to mutation steps (most of the ribotypes are separated by one mutational step but eight mutations separate r4 from r6).

Over the whole dataset (592 individuals), nuclear microsatellite loci displayed moderate to high numbers of alleles, ranging from three to 22 for the loci 6C7 and 7D3, respectively. For the 201 ITS2 sequences, characterised by 24 polymorphic sites and four indels, 21 ribotypes were identified. The most genetically diverse population was found in Chatham Island (Table S1). Observed heterozygosity (Ho) and Allele richness (Ae) were significantly higher in New Zealand than in Chile (Mann and Withney, P<0.05) but no significant differences were encountered for expected heterozygosity (He) and ribotype richness (Re) (Table S1). Allelic and ribotypic richness in Chile (Ae = 0.95±0.42 and Re = 0.52±0.37, Table S1) are half of those observed in New Zealand (Ae = 1.76±0.69 and Re = 0.97±0.77, Table S1), despite more sampling in Chile. The average expected heterozygosity is 25% lower in Chile than in New Zealand (He = 0.32 and 0.44 respectively, Table S1). Moreover, the allele accumulation curve describing the number of alleles observed as a function of sampling effort (Figure S1) demonstrated clearly that the saturation point was reached in Chile but not in New Zealand. It revealed that Chile is a subsample of the New Zealand genetic diversity (84% of the alleles observed in Chile were also found in New Zealand). Similarly, the ITS ribotype r1 which is ubiquitous in Chile is also the most common ribotype in the eastern coast of New Zealand (Table S1, Figure 1). The eleven individuals sequenced for *rbc*L from Chile (CH-MAU, N = 2; CH-ANC, N = 1), New Zealand (NZ-WIN, N = 2; NZ-SCB, N = 1; NZ-PGB, N = 1; NZ-MOU, N = 1) and Chatham Island (NZ-CHT, N = 1) displayed the same haplotype as *G. chilensis* sequences from Chile deposited in GenBank [27], [28]. No significant linkage disequilibrium was found among the five microsatellite loci. Most of the nineteen sampled populations were at H-W equilibrium except two populations from Chile and four populations from New Zealand (Table S1). In the population NZ-MOU, the strong heterozygote excess associated with a high number of repeated multilocus genotypes suggests the occurrence of asexual reproduction. The repeated genotypes of this population were thus removed for the analysis of the genetic structure to avoid erroneous individual assignation and flawed reconstruction of topological relationships among populations. The CH-MOL population also displayed numerous repeated genotypes but this was associated with a very low number of alleles at all loci (Table S1). These repeated genotypes were likely to have originated from distinct sexual reproduction events [14] and thus were included in all analyses.

### Genetic structure

**Figure 2:**
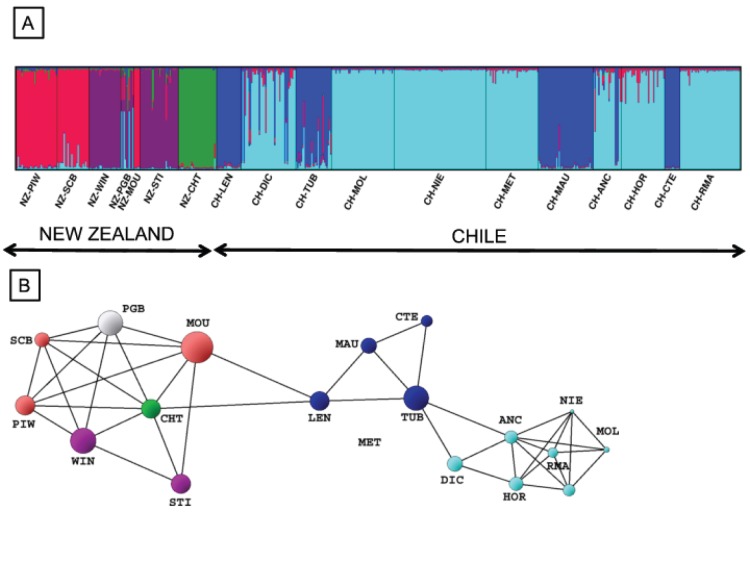
Genetic population structure of New Zealand and Chilean *Gracilaria* populations. (A) based on Bayesian analysis with STRUCTURE software [37], proportions of individual multilocus genotypes were assigned to each of the 5 virtual genetic clusters indicated by the different colours; (B) based on graph analysis with POPULATION GRAPH software [40], differences in node size reflect differences in within population variability whereas edge lengths represent the genetic variation between pairs of samples, there were significantly more edges within New Zealand and Chilean groups than between the two groups (P<0.0001). Node colour corresponds to the 5 clusters as determined by STRUCTURE. For both analyses: N = 7 New Zealand populations, 11 Chilean populations, 567 individuals, 5 codominant loci. Population codes are provided in Table S1.

We found strong evidence for genetic divergence between New Zealand and Chilean populations supported by both model based clustering and graph theory analyses of microsatellite data (Figure 2A and 2B). First, there was a remarkable lack of admixture among populations (Figure 2A). All individuals within a given population were assigned to the same cluster, except for the NZ-PGB population in New Zealand. Second, the topology of the network has 42 edges with two visually identifiable sub-graphs corresponding to the different sides of the South Pacific Ocean (Figure 2B). The two connections between CH-LEN (Chile) and NZ-CHT and NZ-MOU (New Zealand) form the only link between the two continents revealing significant genetic differentiation between New Zealand and Chile (Figure 2B; P<0.0001). Only the POPULATION GRAPH resulting from the exclusion of CH-LEN population located in the Araucanian region reveals two distinct disconnected sub-graphs suggesting that this region could be the unique source of transpacific connectivity. In addition, the Araucanian region hosts the three most genetically diverse populations in Chile for both allele and haplotype richness (i.e. CH-DIC, CH-LEN and CH-TUB; Table S1). In the west Pacific area, populations were highly genetically differentiated based on microsatellite data. Three clear clusters were defined separating the Chatham Islands population from New Zealand populations (Figure 1). Even if populations located in the eastern part of the Cook Strait (NZ-PIW, NZ-SCB and NZ-MOU) were grouped in the same cluster, no clear geographic pattern was observed in New Zealand (Figure 1). In contrast, ITS2 supports a strong Western and Eastern separation of New Zealand populations with highly divergent ribotypes restricted to the three westernmost populations (NZ-WIN, NZ-PGB and NZ-MOU, Figure 1). In Chile, two well-differentiated groups of populations were identified based on microsatellite data analyses but these did not correspond to a geographic arrangement of sampling sites (Figure 2A and 2B). This structure was not seen with the ITS2 owing to the presence of the ribotype r1 at high frequency in all Chilean populations (Figure 1).

### Bottleneck test using microsatellite data

**Figure 3:**
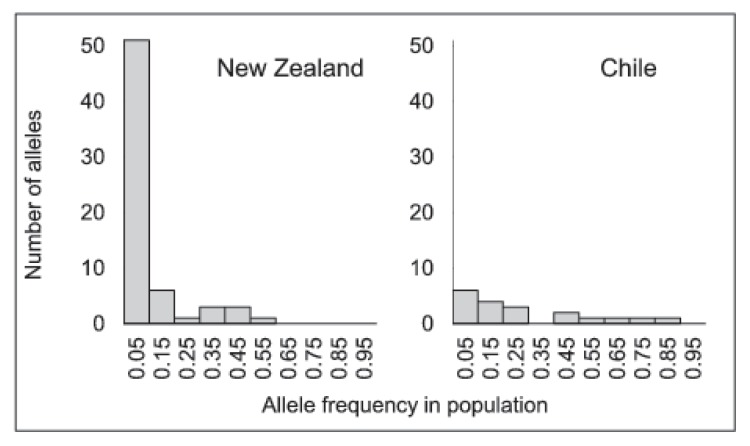
Number of alleles observed in each of the 10 allele frequency classes defined in New Zealand and Chilean populations (pooled data from each side of the South Pacific coast). Reduced number of low frequency alleles is characteristic of a bottleneck [47].

We found evidence of a bottleneck in Chile where rare alleles (alleles with frequency <5%) were 10 times less common than in New Zealand (Figure 3). New Zealand populations as a whole were near mutation drift equilibrium as revealed by the distribution of allele frequency following the characteristic L-shaped distribution (Figure 3). The distribution of allele frequencies in Chile clearly differed from New Zealand even if it did not exactly fit the mode-shifted distribution described by Luikart et al. [41]. However, when the analysis was carried out on each population separately, half of the Chilean populations showed a clear mode-shifted distribution of allele frequencies (5 of the 11 sampled, CH-LEN, CH-NIE, CH-MET, CH-MAU and CH-RMA) while all populations from New Zealand had an L-shaped distribution. Detection of old population bottlenecks is difficult [47], and the possibility that the Chilean bottleneck was not recent enough (>20 generations) could explain the existence of a non-characteristic mode-shifted distribution of allele frequencies.

### Dating of past demographic changes using ITS2

Signatures of demographic expansion were detected in three regions (West New-Zealand, Chatham Island and Chile) by some of our estimators. A significant signature of expansion was observed by Fu’s Fs [42] in Chile (Fs = −5.34, P<0.001), Western New Zealand (Fs = −3.704, P<0.001) and Chatham Island (Fs = −2.373, P = 0.042) but was not significant for Eastern New Zealand (Fs = −1.422, P = 0.187) and New Zealand as a whole (Fs = −1.749, P = 0.398) (Table 1). SSD value and Raggedness index reject the spatial expansion hypothesis only for Eastern New-Zealand (Table 1). Despite partitions, Ramos-Onsins and Rozas’s R_2_ values [43] were low (between 0.0435 and 0.126), which is expected under a scenario of recent demographic expansion, but not significant (Table1). Estimated τ-values under the spatial expansion model were 0.260 in Chile, 1.344 in Chatham Island and 4.833 in West-New Zealand (Table 1). These τ-values indicate that an early wave of expansion took place on the West coast of New Zealand, followed by Chatham Island and then Chile. The almost complete lack of fossils prevents precise calibration of a molecular clock for red algae, thus, we used divergence ITS2 rates already published for plants [46] to estimate the dates of the demographic expansion of *G. chilensis*. Expansion dates were estimated at 360,000 (0–540,000) years for the West coast of New Zealand, 100,000 (54,000–290,000) years for Chatham Island and 19,000 (7,000–45,000) years for Chile based on ITS2 data.

**Table 1:** Tests of demographic changes based on ITS2 sequences.

	South PacificOcean	South West Pacific				South East Pacific Chile
		New Zealand	West NewZealand	East NewZealand$	Chatham	
Sample size	201	93	30	51	12	108
*F*_S_ (P value)	−5.139 (0.05)	−1.749 (0.40)	−3.704 (0.00)	−1.422 (0.19)	−2.373 (0.04)	−5.342 (0.00)
R_2_ (P value)	0.044 (0.11)	0.084 (0.42)	0.091 (0.11)	0.083 (0.26)	0.126 (0.09)	0.043 (0.16)
Spatial Expansion:						
SSD (P value)‡	0.029 (0.51)	0.049 (0.25)	0.004 (0.40)	0.019 (0.00)	0.0003 (0.99)	0.0005 (0.14)
Rag (P value)‡	0.132 (0.72)	0.100 (0.59)	0.359 (0.62)	0.172 (0.04)	0.018 (0.99)	0.359 (0.51)
τ (90% CI)	8.771	7.104	4.833	nc#	1.344	0.260
	(0.303–13.897)	(0.809–12.855)	(0.000–7.295)		(0.728–3.974)	(0.093–0.610)

Departure from neutrality tested using Fu’s Fs [42] and Ramos-Onsins and Rozas’ R_2_ statistic [43]. Significance determined using 10,000 simulated data sets implemented by DnaSP version 5.10.01 [44]. τ-values estimated from demographic expansion models using ARLEQUIN v 3.11 [33]. Goodness of fit tests for a model of population expansion calculated from the sum of squared deviation (SSD) and the Harpending’s raggedness index (Rag). Significance assessed by bootstrapping (10,000 replicates; ARLEQUIN v 3.11) [33].

$ All East New Zealand populations excluding Chatham;

‡ P values of (Expected SSD > Observed SSD) or P (Expected Rag > Observed Rag) superior to 0.05 means the null hypothesis of spatial expansion can’t be rejected;

# nc: τ-value not calculated when spatial expansion is rejected.

## Discussion

### *Gracilaria Chilensis* a non-native species in chile: recent range expansion from new zealand

Both *rbc*L and ITS2 data confirmed that specimens of *G. chilensis* found in New Zealand and Chile are conspecific [25], and revealed that *G. chilensis*, originally described in Chile [48], is paradoxically not native to this country. Several lines of evidence support the hypothesis that *G. chilensis* arrived in Chile as a result of recent trans-oceanic long-distance dispersal from New Zealand. Firstly, G. *chilensis* is the unique representative species of *Gracilaria* on the Chilean coast ([25], this study) whereas four native species are recorded for both New Zealand and southern and southeastern Australia [49], [50], [51]. Secondly, the lack of molecular divergence between sequences from New Zealand and Chile (*rbc*L: only one mitotype encountered; ITS2: the same common ribotype r1 found in New Zealand and Chile) supports the hypothesis of a recent co-ancestry. In Chile, the predominant ribotype r1 is likely to be the ancestral allele since it occupied the centre of the network from which private ribotypes were connected by only one mutational step. In agreement with other phylogeographic studies ([52] and references below), the absence of this predominant ribotype r1 on the west coast of New Zealand suggests that only populations from the east coast participated in the colonisation of Chile. Finally, the Chilean populations contained only a subset of the microsatellite diversity present in eastern New Zealand populations, consistent with a recent range expansion from the western to eastern Pacific. The higher genetic diversity in sites of the Araucanian region (CH-LEN, CH-TUB, CH-DIC) and the location of the node connecting Chile and New Zealand populations in CH-LEN (graph analysis) strongly suggests that the arrival of *G. chilensis* in Chile occurred in the Araucanian region.

Population expansions were detected on both sides of the Pacific, but Chile seems to have experienced demographic changes more recently than New Zealand. The rough estimate of 19,000 years since demographic expansion in Chile is concordant with previous studies of evidence of colonisation of species to Chile from New Zealand at the end of LGM, by rafting along the West Wind Drift [53], [54], [55]. Rafts are formed mainly by intermingled free-floating algae (mostly kelps) but also include a number of invertebrates and other non-buoyant seaweeds species [56]. The two case studies on the red algae *Bostrychia intricata* [54] and *Capreolia implexa* [55] strongly suggest that trans-oceanic dispersal of non-buoyant species has occurred in association with rafting seaweeds. It is likely that *G. chilensis*, a species with the capacity to propagate by free-living fragments, reached Chilean coasts by the same process. Further, it has been argued that this eastward current was probably reinforced and faster at the end of the LGM [57], supporting the idea that transoceanic connectivity was strongest at that period. In addition, contrary to previous studies, archaeological samples of *G. chilensis* found in Monte Verde [11] provide evidence that this species was present in Chile 14,600 years ago, reinforcing the hypothesis that the transoceanic dispersal occurred at least at the end of LGM period.

### *Gracilaria chilensis* establishment and expansion in chile

Successful establishment and expansion of *G. chilensis* was probably facilitated by intrinsic characteristics such as phenotypic plasticity and life history traits, conferring capacity to live in new environments [58]. Such characteristics, particularly tolerance of a wide range of abiotic conditions, have been reported to have contributed to the establishment of the invasive species *G. vermiculophylla* (Ohmi) Papenf. [59] in estuaries and muddy bays. A common characteristic of *Gracilaria* sp., reported as undergoing rapid range expansion, is the formation of extensive mats in muddy bays by vegetative fragmentation [14], [50], [59]. Its capacity for clonal propagation through thallus fragmentation and its high tolerance to adverse physical conditions allows *G. chilensis* to survive for long periods out of the water, enabling transportation and dispersal. Transport and exchanges of this alga are active between coastal communities of fishermen and have contributed to a very recent expansion in the range of distribution of this species in Chile [14]. Indeed, new farms were planted in the region of Atacama (27°S) in the early 1980s, at distances up to 650 km from the northernmost known natural population [48], [60]. Moreover, exchanges over smaller distances have also been reported in southern Chile and genetic analyses have shown that particular genotypes have been gradually passed from one farm to another through the exchange of thallus fragments used to reseed artisanal farms [14].

Modern anthropogenic transport of living *G. chilensis* has been documented to have deeply affected the distribution range of the species and the genetic structure of populations [14], [18], [19]. As archaeological data has provided evidence that the species was used by early human communities as food or medicine, one can wonder whether anthropogenic transport was relevant at that time. Since the species was found in Monte Verde, a site located approximately 60–70 km from the sandy coast and estuaries during the late Pleistocene, archaeologists have hypothesised that humans were travelling to the coast or exchanging goods with coastal inhabitants, deliberately transporting marine species [11], [61]. However, the role of early human transport and/or exploitation of these marine species on the establishment success, in particular of *G. chilensis*, remains an open question that could be further addressed with more powerful markers.

### Effect of human practices on genetic resources: an exceptionally low diversity in Chile

The signature of bottleneck was observed with the typical shift of the microsatellite allele frequency distribution and could explain the strikingly low genetic diversity in Chile when compared to New Zealand. Because this distortion is transient and likely to be detectable for only a few dozen generations [41], this bottleneck is likely to be recent, although it is difficult to determine if it is a signature of the overexploitation of the early 1980s [18] or of the intensive cultivation of the late 1990s [14].

Domestication is a process that usually lasts up to several millennia as a result of a mostly unconscious human selection (at least in the early stages), leading to complex evolutionary trajectories associated with changing selective pressures across the history of human utilisation/cultivation [62]. Such effects of unconscious human selection have been observed for *G. chilensis* cultivated in Chile, with a modification of life history traits [14]. In contrast, a demographic bottleneck associated with overexploitation is a transient, relatively short-term process. In this context, even if the relative influence of overharvesting and domestication cannot be accurately quantified, it is possible to infer that domestication, through selection of clonal strains, is a long-lasting process that may have limited the replenishment of genetic diversity in natural populations in Chile. This loss of genetic diversity due to clonal propagation has been documented for many domesticated species [15], [16]. However, traditional complex culture practices of land plants actively reintroduce genetic diversity through maintenance of some sexual reproduction [15]. In the case of *G. chilensis*, the erosion of genetic diversity along the Chilean coast may have seriously limited the capacity of individuals to adapt to changing environments and/or to respond to further human selective pressure for aquaculture needs. In conclusion, this study emphasises how the conjunction of recent bottleneck with the predominance of clonal propagation seems to be driving this species into an extinction vortex in Chile.

## Supporting Information

Figure S1 **Comparison of allele accumulation curves between Chilean and New Zealand (including Chatham Island) samples.**
(PDF)

Table S1 **Sampling locations and diversity measures for microsatellites (5 loci) and ITS2 sequences in** ***G. chilensis.*** N, number of individuals per sampling locations used for microsatellite genotyping; (Ho) observed heterozygosity, (He) expected heterozygosity, Ae, multi-locus estimates of expected allelic richness based on the smallest sample size (20 genes/10 individuals); Pa: number of private alleles; Weir and Cockerham’s [32] *F*IS estimate (NS non-significant, * significant deviation from HW expectation, *P*<0.0028, α = 0.05, Bonferroni correction of multiple test with 18 populations); n, number of individuals used for sequencing; nR, number of ribotypes; Re, expected haplotype richness based on the smallest sample size (5 ribotypes); Ribotypes: details of the different ribotypes observed in the populations, private ribotypes are in bold. Mean ± SEM are indicated for all New Zealand samples (i.e. Global New Zealand) and all Chilean samples (i.e. Global Chile).
(PDF)
