# Supplementary material for: Tracing the Trans-Pacific Evolutionary History of a Domesticated Seaweed (Gracilaria chilensis) with Archaeological and Genetic Data
Source: PLoS One. 2014 Dec 11;9(12):e114039. doi: 10.1371/journal.pone.0114039 (PMC4263469; doi:10.1371/journal.pone.0114039)
Supplement: Figure S1 — Comparison of allele accumulation curves between Chilean and New Zealand (including Chatham Island) samples. (PDF) [file pone.0114039.s001.pdf]

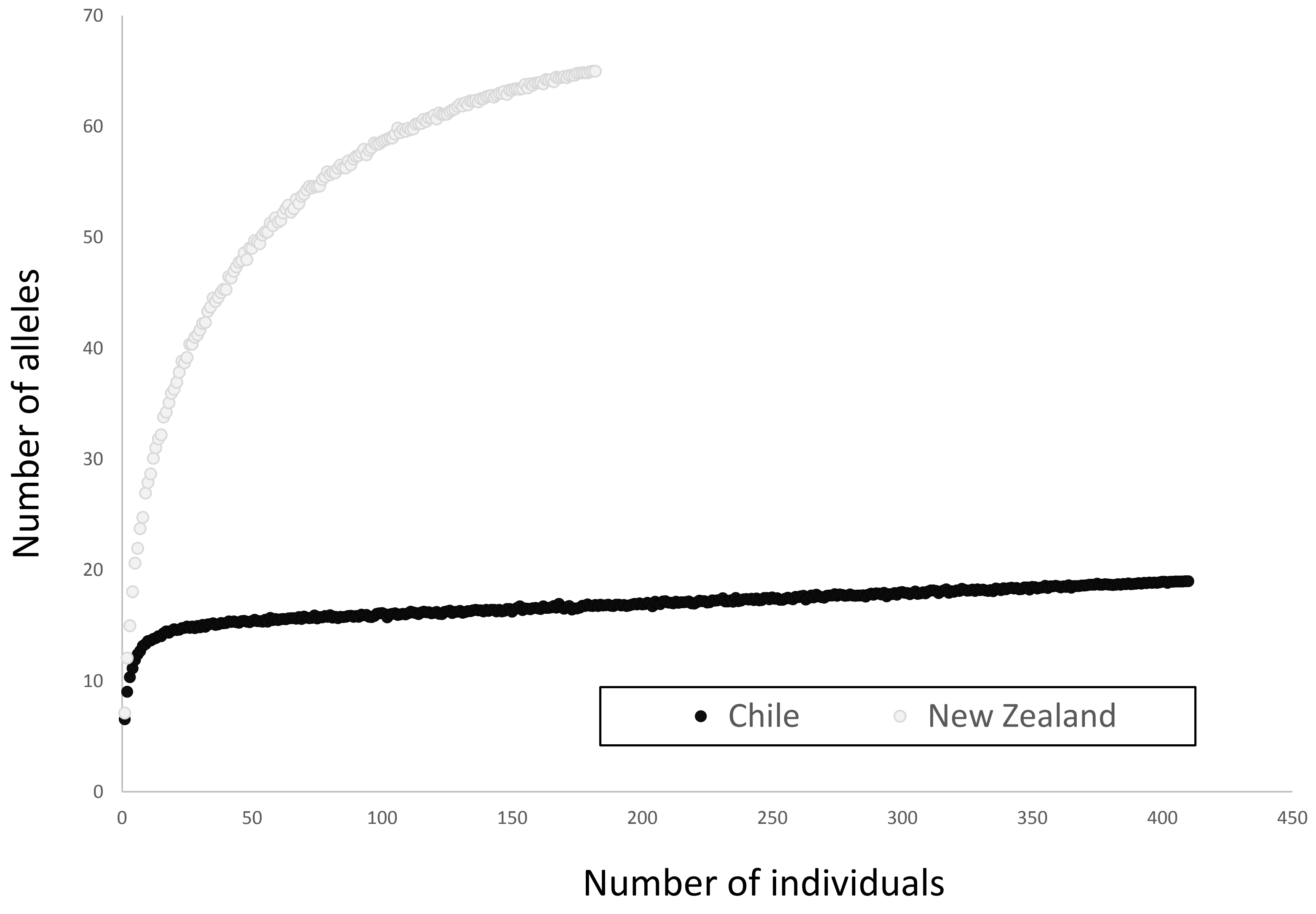

FigureS1: Comparison of allele accumulation curves between Chilean and New Zealand (including Chatham Island) samples.
