# Supplementary material for: Tracing the Trans-Pacific Evolutionary History of a Domesticated Seaweed (Gracilaria chilensis) with Archaeological and Genetic Data
Source: PLoS One. 2014 Dec 11;9(12):e114039. doi: 10.1371/journal.pone.0114039 (PMC4263469; doi:10.1371/journal.pone.0114039)
Supplement: Table S1 — Sampling locations and diversity measures for microsatellites (5 loci) and ITS2 sequences in G. chilensis. N, number of individuals per sampling locations used for microsatellite genotyping; (Ho) observed heterozygosity, (He) expected heterozygosity, Ae, multi-locus estimates of expected allelic richness based on the smallest sample size (20 genes/10 individuals); Pa: number of private alleles; Weir and Cockerham’s [32] FIS estimate (NS non-significant, * significant deviation from HW expectation, P<0.0028, α = 0.05, Bonferroni correction of multiple test with 18 populations); n, number of individuals used for sequencing; nR, number of ribotypes; Re, expected haplotype richness based on the smallest sample size (5 ribotypes); Ribotypes: details of the different ribotypes observed in the populations, private ribotypes are in bold. Mean ± SEM are indicated for all New Zealand samples (i.e. Global New Zealand) and all Chilean samples (i.e. Global Chile). (PDF) [file pone.0114039.s002.pdf]

Table S1: Sampling locations and diversity measures for microsatellites (5 loci) and ITS2 sequences in *G. chilensis*. N, number of individuals per sampling locations used for microsatellite genotyping; (Ho) observed heterozygosity, (He) expected heterozygosity, Ae, multi-locus estimates of expected allelic richness based on the smallest sample size (20 genes / 10 individuals); Pa: number of private alleles; Weir and Cockerham's [32] F_{IS} estimate (NS non-significant, * significant deviation from HW expectation, $P < 0.0028$, $\alpha = 0.05$, Bonferroni correction of multiple test with 18 populations); n, number of individuals used for sequencing; nR, number of ribotypes; Re, expected haplotype richness based on the smallest sample size (5 ribotypes); Ribotypes: details of the different ribotypes observed in the populations, private ribotypes are in bold. Mean ± SEM are indicated for all New Zealand samples (i.e. Global New Zealand) and all Chilean samples (i.e. Global Chile).

		Microsatellites						ITS2 sequences			
Population (abbreviation)	Position	N	Ae	Pa	Ho	He	F_{IS}	n	nR	Re	Ribotypes
**NEW ZEALAND**											
**West New Zealand**											
Whanganui Inlet (NZ-WIN)	40° 34' S, 172° 32' E	25	1.84	1	0.28	0.44	0.37*	11	2	0.45	r6, r8
Golden Bay (NZ-PGB)	40° 48' S, 172° 48' E	10	2.65	3	0.39	0.56	0.35*	8	2	0.62	r6, r7
Moutere Inlet (NZ-MOU)	40° 59' S, 173° 01' E	30	0.86	2	0.59	0.33	- 0.78*	11	3	0.91	r6, r9, r10
**East New Zealand**											
Pautahanui Inlet (NZ-PIW)	41° 06' S, 174° 49' E	32	2.32	11	0.56	0.52	- 0.07^{NS}	14	2	0.60	r1, r4
Scorching Bay (NZ-SCB)	41° 18' S, 174° 48' E	25	0.82	0	0.27	0.23	- 0.18^{NS}	15	3	1.18	r1, r2, r3
Avon-Heathcote estuary (NZ-CAH)	43° 36' S, 172° 45' E	-	-	-	-	-	-	5	2	1.00	r1, r4
Stewart Island (NZ-STI)	46° 54' S, 168° 07' E	30	1.97	7	0.50	0.48	- 0.02^{NS}	17	2	0.29	r1, r5
**Chatham Island**											
Chatham Island (NZ-CHT)	43° 52' S, 176° 33' W	30	1.86	6	0.19	0.49	0.62*	12	7	2.74	

											r1, r11, r12, r13, r14, r15, r16
**GLOBAL NEW ZEALAND**		182	1.76 ± 0.69	49/69	0.40 ± 0.16	0.44 ± 0.12	0.21*	93	16	0.97 ± 0.77	15/21
**CHILE**											
**Araucanian region**											
Dichato (CH-DIC)	36° 45’ S, 73° 11’ W	43	1.29	0	0.32	0.38	0.17 ^{NS}	13	2	0.64	r1, r21
Lenga (CH-LEN)	36° 32’ S, 72° 56’ W	19	1.32	0	0.41	0.39	- 0.02 ^{NS}	9	2	0.56	r1, r17
Tubul (CH-TUB)	37° 15’ S, 73° 26’ W	28	1.72	0	0.54	0.58	0.10 ^{NS}	9	3	1.11	r1, r20, r21
**Valdivia river región**											
Molinos (CH-MOL)	39° 50' S, 73° 23' W	49	0.63	0	0.30	0.23	- 0.28 *	12	2	0.84	r1, r18
Niebla (CH-NIE)	39° 52' S, 73° 23' W	72	0.44	0	0.13	0.13	0.00 ^{NS}	9	1	0.00	r1
**Austral region**											
Metri (CH-MET)	41° 36’ S, 72° 42’ W	41	0.65	1	0.37	0.30	- 0.24 ^{NS}	8	2	0.62	r1, r21
Maullin (CH-MAU)	41° 37’ S, 73° 35’ W	43	1.26	0	0.48	0.46	- 0.04 ^{NS}	7	2	0.71	r1, r19
Ancud (CH-ANC)	41° 52' S, 73° 48' W	22	1.23	0	0.31	0.36	0.17 ^{NS}	9	2	0.56	r1, r21
Hornopiren (CH-HOR)	41° 58' S, 72° 28' W	34	0.69	0	0.19	0.26	0.27*	12	2	0.68	r1, r21

Chaiten (CH-CTE)	42° 55' S, 72° 42' W	12	0.63	0	0.23	0.20	- 0.14 ^{NS}	9	1	0.00	r1
Raul Marin (CH-RMA)	43° 46’ S, 72° 57’ W	47	0.60	0	0.24	0.25	0.06 ^{NS}	11	1	0.00	r1
**GLOBAL CHILE**		410	0.95 ± 0.42	4/69	0.32 ± 0.12	0.32 ± 0.13	0.04 ^{NS}	108	6	0.52 ± 0.37	5/21
